# Targeting the invisible: precision fiducial marker placement in poorly visible liver tumors prior to percutaneous ablation using real-time image fusion guidance

**DOI:** 10.3389/fradi.2025.1659739

**Published:** 2025-11-20

**Authors:** N. Villard, G. Tsoumakidou, F. Gay, P. Rousset, G. Passot, A. Muller, J. Dumortier, P. J. Valette, L. Milot

**Affiliations:** 1 Department of Diagnostic and Interventional Radiology, Lausanne University Hospital and University of Lausanne, Lausanne, Switzerland; 2 Department of Radiology, Hospices Civils de Lyon, Lyon University Hospital, Lyon, France; 3 Department of Digestive Surgery, Hospices Civils de Lyon, Lyon University Hospital, Lyon, France; 4 Fédération des Spécialités Digestives, Hôpital Edouard-Herriot, Hospices Civils de Lyon, Lyon University Hospital, Lyon, France

**Keywords:** image fusion, fiducial markers, liver neoplasms, image-guided procedures, neoplasm localization

## Abstract

**Purpose:**

This study aimed to assess the feasibility and accuracy of fiducial marker placement using US-CT/MRI fusion imaging guidance in poorly conspicuous liver tumors prior to percutaneous thermal ablation (PTA).

**Method:**

From January 2016 to February 2018, 30 consecutive patients with 38 liver lesions that were poorly or not visible on conventional ultrasound underwent fiducial marker placement under real-time US-CT/MRI fusion imaging before the PTA procedure. Marker position was confirmed via CT or MRI immediately after placement. The shortest distance between the marker and the edge of the target lesion, the lesion size, and the depth were measured. The fiducial marker placement was considered successful if the marker was within, in contact or ≤5 mm distance from the lesion; a distance >5 mm was considered a failure.

**Results:**

Of the 38 lesions, 28 (74%) were undetectable using ultrasound alone, while 10 (26%) were not confidently identified. After fusion, 26 lesions (68%) showed enhanced visibility, while 12 (32%) remained undetectable. Overall, the mean distance between the fiducial marker and the lesion's edge was 4 mm (range: 0–45 mm). Successful placement was achieved in 30 lesions (79%): 27, inside or in contact, and 3, at a <5 mm distance from the target lesion. Placement was unsuccessful in eight lesions (21%). No procedure-related complications occurred.

**Conclusions:**

The present work suggests that pre-PTA placement of a fiducial marker in poorly visible tumors using real-time US-CT/MRI fusion imaging is accurate, potentially enhancing the effectiveness of subsequent PTA.

## Introduction

Primary and secondary liver malignancies are among the most common and clinically significant tumors worldwide, representing a major cause of cancer-related morbidity and mortality (1–3). Hepatocellular carcinoma (HCC) and liver metastases, particularly from colorectal cancer, are frequently encountered in clinical practice and often necessitate locoregional treatment strategies. The 2025 European Society for Medical Oncology (ESMO) clinical practice guidelines recommend percutaneous thermal ablation (PTA) along with surgery for very early [Barcelona Clinic Liver Cancer (BCLC)-0] and early (BCLC-A) HCC patients who are not candidates for liver transplantation (4). In a similar setting, the results of the COLLISION trial indicate that PTA can even represent the standard of care for small liver metastases from colorectal cancer (5–11).

Imaging plays a central role in PTA, both for treatment planning and guidance during the procedure itself. Among imaging modalities, contrast-enhanced magnetic resonance imaging (MRI) offers the highest sensitivity for liver tumor detection, followed by multiphase contrast-enhanced computed tomography (CECT) (12, 13). Despite its superior lesion visibility, MRI guidance remains limited in clinical practice due to equipment availability, higher cost, and the need for MR-compatible interventional tools. CT “step-and-shoot” guidance is widely used for percutaneous procedures but has limitations due to the single axial plane view and the lack of real-time feedback during needle progression. Additionally, because liver enhancement after contrast media administration is transient, lesion visibility may be limited to a short time window, making consistent targeting during needle placement more difficult (14, 15).

Ultrasound (US) is the preferred image-guidance modality for many interventional radiologists due to real-time, multiplanar capabilities, wide availability, and low cost. However, its sensitivity is limited, particularly for lesions of small size, located deep in the liver (i.e., liver dome) or obscured by factors such as the presence of background macronodular cirrhosis or heterogeneous liver echostructure. To overcome these limitations, real-time image fusion of US with pre-acquired CT or MRI datasets has emerged as an efficient technique, enabling better lesion localization and improving the feasibility of image-guided interventions for their detection by radiologists (12–14).

The use of tumor landmarking with small fiducial markers has been employed in the management of potentially operable liver metastases at risk of becoming radiologically occult following neoadjuvant chemotherapy (15). When placed percutaneously before initiation of chemotherapy, these markers have proven useful for precise localization of the initial tumor site, facilitating precise surgical resection or thermal ablation—even in cases of complete tumor disappearance (16–18). Fiducial markers are also commonly used to enable accurate lesion tracking during stereotactic body radiation therapy (SBRT) (21). Their visibility across all imaging modalities—including US, contrast-enhanced CT, and MRI—makes them a reliable tool for long-term lesion tracking and interventional planning.

Treating tumors that are occult or poorly visible on ultrasound and/or CT with PTA can be particularly challenging. Haochen et al. (20) already described the use of MR-guided fiducial implantation before PTA. In the present study, we describe a two-step strategy to facilitate the targeting of occult lesions by the percutaneous insertion of a small metal fiducial marker in these tumors under real-time fusion imaging guidance prior to PTA. Shortly after, the fiducial placement is confirmed with CT or MRI. This study aims to evaluate the feasibility and precision of such fiducial marker placement guided by fusion imaging.

## Methods

Our institutional review board approved this retrospective study and waived written informed consent for the review of images and records.

### Study population

Out of a total of 198 patients treated from January 2016 to February 2018 in our tertiary reference care center, 30 consecutive patients who had a fiducial marker inserted prior to PTA were included in our series. All patients presented with malignant tumors, proven either by pathology or based on the Liver Imaging Reporting and Data System (LI-RADS) criteria for HCC (19), and the decision to treat with PTA was taken in a multidisciplinary cancer board meeting, including radiologists, interventional radiologists, radio-oncologists, oncologists, hepatobiliary surgeons, and gastroenterologists. Inclusion criteria were all liver tumors visualized on CECT and/or MRI and invisible/not clearly detectable on ultrasound. Exclusion criteria were tumors clearly visible under US guidance.

In accordance with our patient management protocol, all the patients were seen in the pre-intervention clinic 2 weeks prior to treatment by a senior interventional radiologist. This consultation included an ultrasound examination and a re-reading of the scanner and/or MRI to establish whether each lesion to be treated had sufficient visibility for a safe PTA targeting under US or CT guidance. When the target lesion was deemed challenging to visualize (small size, deep location, or heterogeneous liver parenchyma), the placement of a fiducial marker guided by image fusion was proposed to the patient. After informed consent, the procedure was organized in the period preceding the PTA.

### US fusion imaging workup and percutaneous procedure

The fiducial marker placements were performed by one of two senior interventional radiologists (30 and 10 years of experience in interventional abdominal radiology, respectively). Before the procedure, routine coagulation values were checked. Fusion was obtained using the software available on our ultrasound systems (Aplio 500, Toshiba Medical Systems Corp., Tokyo, Japan; EPIQ 7, Philips Healthcare, Andover, MA, USA). The 18 G 137-mm-long marker applicator needle (O-Twist Marker, BIP GmbH, Türkenfeld, Germany), initially commercialized for breast tissue marking, was inserted under local anesthesia and aseptic conditions.

All fiducial placement procedures were performed under local anesthesia using strict aseptic technique. The interventional radiologist selected the most appropriate MRI or CT series—typically the late arterial phase for HCC and the portal venous phase for metastases—to optimize visualization of both the tumor and key vascular structures used as anatomic landmarks for liver lesion mapping and image fusion. The point-to-point registration technique was utilized for image fusion. Manual adjustments were made as needed, guided by the main vascular landmarks, especially during the transition from anterior subcostal to lateral intercostal ultrasound window. If the tumor was peripheral, and especially if the lesion remained undetectable even after fusion, the adjustment focused on replicating the peripheral intrahepatic vascular tree near the tumor, as shown on the CT or MRI. Then, whether the lesion was optimally seen, doubtful, or undetectable, the progression of the needle was followed under real-time US guidance to the area of interest as determined by the fused images, and the marker was implanted.

Immediately following the fiducial marker placement, a CT or MRI with protocols optimized for tumor detection was performed. The imaging protocol was adapted to ideally identify the target lesion and its proximity to the fiducial marker. Any complications related to the procedure were systematically recorded.

### Imaging analysis

All the images were retrospectively analyzed in consensus by a senior radiologist and a junior radiologist blinded to the clinical context. The size and segment location of the tumors in the liver were reported from the initial CT or MRI. The distance from the marker applicator entry point at the skin to the tumor, the echostructure of the underlying parenchyma (normal, heterogeneous, nodular), and the level of lesion conspicuity (undetectable, doubtful, optimal), were reported from fusion (ultrasound with CT or MRI).

Fiducial marker placement was evaluated on CT or MRI imaging performed immediately after insertion. Measurements included the distance between the marker and the border of the target lesion, the lesion's longest diameter, and its depth (defined as the distance between the skin and the lesion along the planned trajectory for the PTA). Image analysis was performed using the Centricity and AW imaging reading software (GE HealthCare, Waukesha, WI, USA). Placement was deemed successful if the fiducial marker was inside, in contact, or within <5 mm from the lesion border. A distance >5 mm was considered a placement failure. The 5 mm threshold was chosen to be an acceptable distance for a location before ablation.

### Endpoints

The primary endpoint was to assess the accuracy of fiducial marker placement under ultrasound with real-time fusion imaging in cases of invisible or poorly visible liver lesions.

## Results

A total of 30 patients (mean age 64 years, range 40–80 years; 23 males, 7 females) with 38 lesions were included in the study. Twenty-one patients had HCC, and nine patients had liver metastases from colorectal cancer (7), pancreatic cancer (1), and ovarian cancer (1). The mean lesion size was 13 mm (range 4–27 mm). Two lesions were <6 mm, 13 were between 6 and 10 mm, 18 were between 11 and 20 mm, and 5 were >20 mm. Twenty-five lesions were located at the deep superior or posterior parts of the liver, partially hidden by lung, scars from previous treatment, or interposed bowel structure (segments I, VI, VII, and VIII), and 13 lesions were located in regions more accessible to US detection (segments II, III, and V) (Table 1). The mean depth of the lesions was 7 cm (range 3–14 cm).

**Table 1:** Characteristics of the liver lesions and results of fiducial marker placement.

Location of liver tumors	Liver segments	Tumor depth	Tumor size	US tumor conspicuity before fusion	Causes of undetectably needing the use of fusion (several possible)	Tumor conspicuity after fusion	Fiducial marker placement, success = 0–5 mm
Deep segments 25/38 (66%)	VIII (13), VII (7), VI (4), I (1)	*m* = 8 cm (8–14)	7–22 mm	Undetectable (19), Doubtful (6)	Hidden area (11), Small size (6), Liver echo texture (16)	Undetectable (10), Doubtful (3), Certain (12)	Successful (18), Unsuccessful (7)
Superficial segments 13/38 (34%)	V (3), IV (6), III (2), II (2)	*m* = 5 cm (3–8)	4–27 mm	Undetectable (9), Doubtful (4)	Hidden area (2), Small size (4), Liver echo texture (7)	Undetectable (2), Doubtful (3), Certain (8)	Successful (12), Unsuccessful (1)

**Figure 1:**
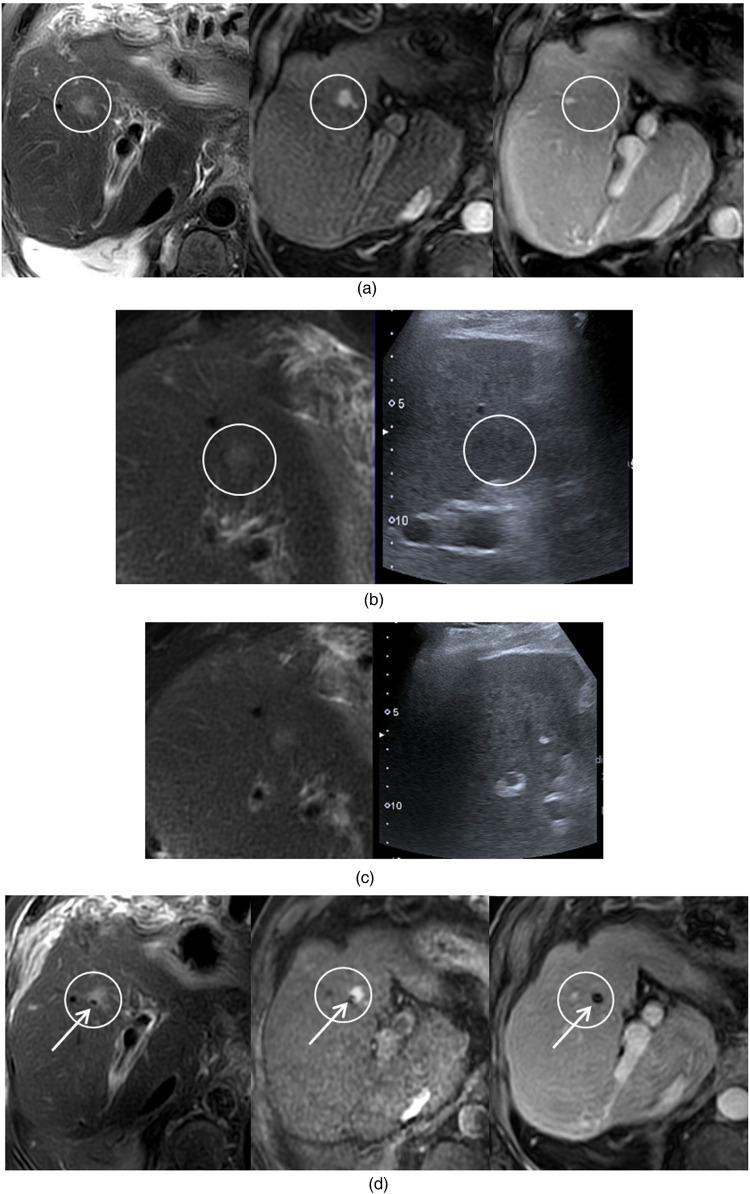
Cirrhotic patient with HCC scheduled for thermal ablation. A second small nodule was identified in the right liver during pre-procedural imaging. The objective was to localize this second nodule for percutaneous treatment during the same session. Lesion detection: **(a)** An 8 mm nodule is visualized on T2-weighted and T1 arterial-phase MRI, but not on T1 portal-phase imaging. **(b)** Fusion imaging was performed with T2 MRI. Due to anterior bowel interposition, an oblique intercostal approach was required. The nodule appeared as a slightly hypoechoic focus on ultrasound, although without certainty. Lesion localization: **(c)** The fiducial marker (white arrow) was placed under combined ultrasound and MR T2 fusion guidance. **(d)** Immediate post-procedural MRI confirmed the appropriate positioning of the marker in close contact with the nodule.

Among the 38 lesions, 28 (74%) were undetectable using ultrasonography alone, and 10 (26%) were doubtful. With the help of the fusion, 26 (68%) lesions showed enhanced visibility, although with unclear boundaries in six cases (Figure 1), while 12 (32%) remained undetectable. The major reasons behind no or poor detection were liver heterogeneity and deep localization (region not accessible by ultrasound). Technically, fusion was primarily useful to distinguish between tumors and neighboring parenchymal heterogeneity or to find an angle of the US beam allowing for a better visualization in cases of deep localization or a hidden part of the liver. All 10 lesions of doubtful detection because of the heterogeneity of the hepatic parenchyma were more distinctly detected. Six lesions located at the very high or posterior part of the right lobe of the liver became visible by adapting the angle of the ultrasound beam according to the fused images.

**Figure 2:**
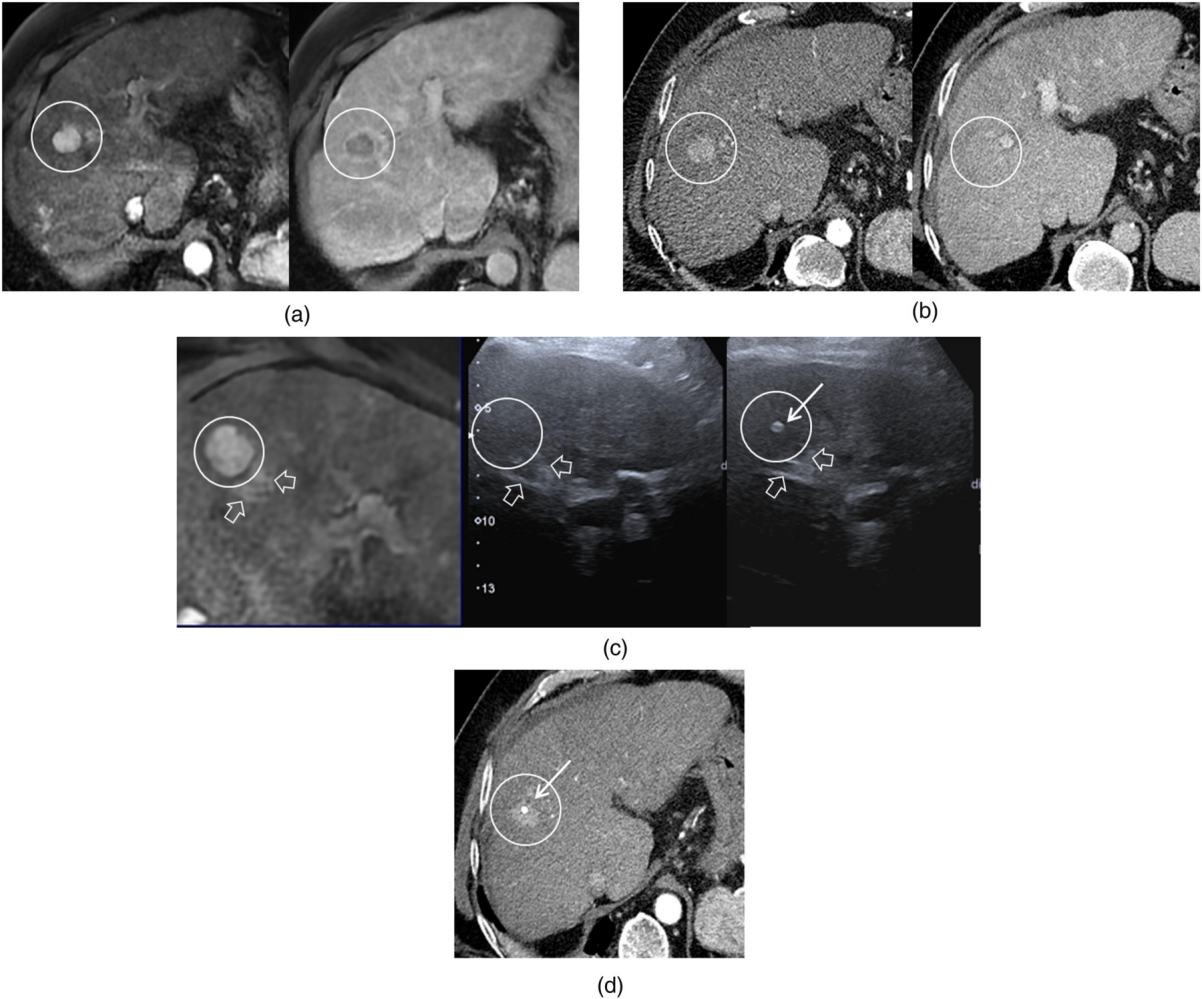
Hepatocellular carcinoma scheduled for thermal ablation. Lesion detection: **(a)** A 20 mm nodule is clearly seen on T1 MRI during both arterial and portal phases. **(b)** On CT, the lesion is detected at the arterial phase but not at the portal phase, highlighting potential difficulties for accurate targeting under CT guidance alone. Lesion localization: **(c)** Fusion imaging with T1 MRI arterial phase failed to directly visualize the nodule on ultrasound. However, adjacent vascular landmarks (hollow arrows) were concordant between MRI and ultrasound, allowing placement of the fiducial marker (white arrow) under ultrasound guidance by targeting the corresponding region. **(d)** Immediate post-procedural CT confirmed satisfactory marker positioning within the tumor volume.

Overall, the mean distance between the tumor and the fiducial marker was 4 mm (range 0–45 mm). For 30 (79%) lesions, the fiducial marker was successfully placed inside or in contact (27/30) or within <5 mm from the target lesion (3/30). The criterion for success for fiducial marker placement was not satisfied for 8 (21%) lesions. For those failures, the distances between the fiducial marker and the lesion border were 7, 8, 10, 15, 15, 20, 22, and 45 mm on control imaging. These cases were related to the lesion depth and insufficient length of the applicator to place the marker adequately (two cases) or to fusion imprecision (six cases). This inaccuracy was observed in 5 (42%) out of the 12 lesions invisible on ultrasound despite fusion. Interestingly, among these 12 lesions totally invisible to US even after fusion, 7 (58%) had a successful fiducial marker placement (Figure 2).

**Figure 3:**
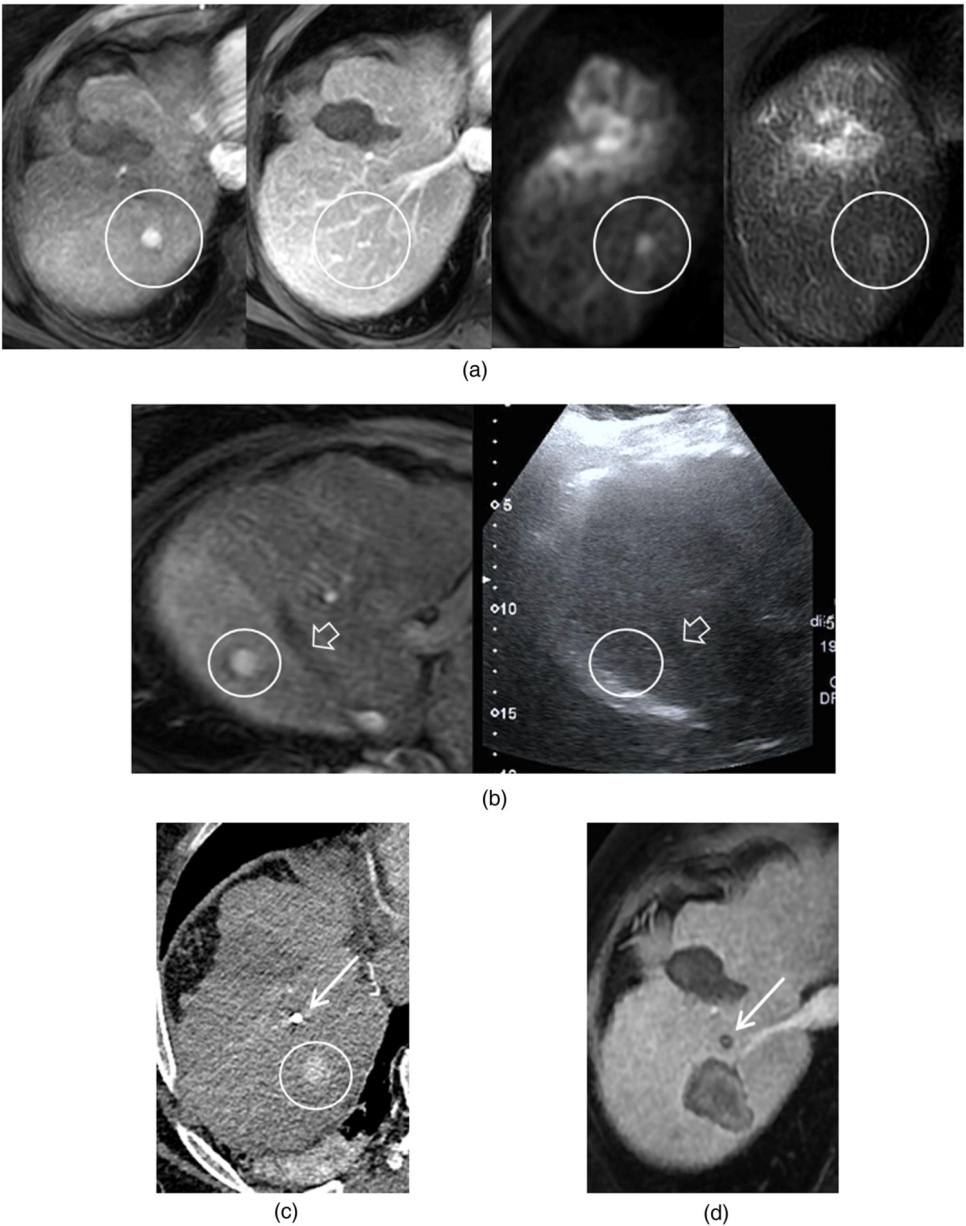
Small recurrent hepatocellular carcinoma occurring 6 months after initial thermal ablation of a first lesion. Lesion detection: **(a)** A 10 mm nodule is identified adjacent to the ablation scar on T1 arterial-phase MRI, T2, and diffusion-weighted (b1000) imaging. It is not visible on portal-phase imaging. Lesion localization: **(b)** Fusion imaging with arterial-phase MRI did not allow confident visualization of the nodule. A fiducial marker was therefore placed under ultrasound guidance, posterior to the right hepatic vein (hollow arrow), in the region corresponding to the tumor on MRI. Due to poor echogenicity, the marker was not visualized on ultrasound after withdrawal of the applicator needle. **(c)** Immediate post-procedural CT showed the marker (white arrow) positioned approximately 4 cm anterior to the tumor. Despite this suboptimal placement, ablation could be performed under CT guidance by targeting posterior to the marker. **(d)** One-month follow-up MRI demonstrated an ablation zone encompassing the tumor, which was no longer visible.

No complication directly or indirectly related to the fiducial marker placement was recorded (Figure 3).

## Discussion

Potentially curative treatment of primary or metastatic liver tumors requires a comprehensive treatment of all liver lesions, combining liver resection and/or PTA (RFA or microwave ablation (MWA)) (3, 7, 8, 16, 20). With thermal ablation, the aim is to obtain complete tumor destruction and to avoid complications related to damage to the heat-vulnerable organs in the vicinity, such as the bile ducts for central tumors, bowel, or even lung for subcapsular tumors. To overcome these difficulties, the tumor must be precisely targeted by the ablation needle, and the ablation parameters must be carefully adjusted according to the size and location of the tumor in relation to surrounding structures. For these reasons, precise localization of liver lesions is crucial for accurate targeting.

Ultrasound is the modality of choice for interventional procedures in the liver due to several advantages, mainly real-time and multiplanar capabilities. In contrast, it is operator dependent, and its sensitivity for detecting malignancies is lower than that of CT or MRI (21, 22). Bae et al. (23) reported that percutaneous RFA was infeasible in approximately one-fourth of patients with metastatic colorectal cancer. The reason for this infeasibility was mainly an unfavorable tumor location and its invisibility on planning US. Lesion visualization can be improved using US contrast (SonoVue, Bracco, Italy), but the enhanced visibility does not last long after the injection and fails to provide guidance during the whole procedure, particularly for hypervascular tumors such as HCC.

Owing to these limitations, PTA under US fusion imaging guidance has been proposed (24, 25). Image fusion software can be used to superimpose the CT or MRI datasets, clearly displaying the lesions onto real-time ultrasound images in order to make the identification of sonographically occult tumors possible (26). Fusion devices often use electromagnetic fields around the patient to locate the US transducer and reformat the corresponding CT or MR images. These images are reformatted through multiplanar algorithms and are displayed in real time alongside the US image, thus enhancing targeting. The incidence of mistargeting after fusion imaging-guided radiofrequency ablation (RFA) appears very low in the literature, as low as 1.3%, according to Lim et al. (27), but up to 4.4% in ultrasound invisible lesions according to Mauri et al. (28). Recently, in a series of 40 liver metastases to be treated by PTA, Hakime et al. (29) showed a significant increase of the tumor conspicuity after localization on fusion imaging, offering a technical success rate of 83% (33/40) in the intention-to-treat analysis and 100% in an analysis of treated tumors. Such papers will certainly, and rightly, contribute to generalizing image fusion for PTA imaging guidance. But with no mention of the effective reliability of the fusion of images in terms of distance from the targeted lesion, specifically when the lesions remain totally invisible to US despite the fusion, they lack an objective measurement of the anatomical precision obtained.

Our series objectively measures the distance between liver tumors that are poorly or not visible to ultrasound and the position of a needle tip placed under image fusion, thanks to the deposition of a marker whose location was verified by CT or MRI. It demonstrates the feasibility and efficiency of fiducial marker placement under fusion. With an average tumor—fiducial marker distance of 4 mm and successful placement in 8 out of 10 cases, image fusion can be considered reliable for tumor localization before PTA. This is remarkable because five of these lesions remained totally invisible on ultrasound, even with the aid of fusion, and the fiducial marker placement relied solely on the fusion technique.

Fiducial markers are commonly placed by the breast radiologists daily for targeting lesions during ultrasound studies or guided biopsies. This technique has become widely accepted to help the surgeon by facilitating and guiding intraoperative tumor resection procedures (30, 31). In US, fiducial markers appear hyperechoic with posterior acoustic shadowing; in MRI, they produce a small blooming artifact; and in CT images, they are clearly visualized as hyperdense structures.

The second key contribution of our series is to confirm the interest of preoperative tracking of liver tumors potentially difficult to detect during PTA by fiducial markers. Once a fiducial marker is adequately placed inside or in close contact with the liver tumor, any technique (US, CT, or both combined) may be employed to guide the ablation needle in a very simple way, similar to cases of lesions well seen on routine imaging. In our practice, ablations are performed under dual guidance; ultrasound to monitor the needle progression, and CT is used to confirm the final position of the needle and to evaluate the outcome of the thermal ablation. In case of tumors completely invisible or poorly visible by either of the two techniques, the fiducial marker greatly facilitates the placement of the ablation needle regardless of the access route imposed by the tumor location and adjacent structures to be preserved.

There remains, however, the question of whether fiducial marker placement should be performed for all cases of tumors that are difficult to locate and proposed for thermal ablation or only for some of them. If the tumor is finally well identified, thanks to the fusion of images as it was for 2/3 of our lesions, the fiducial marker may not be necessary, and the positioning of the ablation needle could be performed directly under fusion during treatment. If, on the contrary, the tumor remains completely invisible on ultrasound despite image fusion, placing a fiducial marker before ablation appears valuable to verify its position at CT or MRI. In the best cases, the fiducial markers will be perfectly in place; in the other cases, the distance from the fiducial marker to the tumor can be appreciated and considered for the positioning of the ablation needle. We recommend fiducial marker placement in all cases of metastases that are at risk of disappearing after chemotherapy and for which lesion-site excision is being considered following neoadjuvant treatment.

This study has several limitations. First, it was a retrospective, single-center study with interventional radiologists highly trained in image fusion and guided puncture, which may limit generalizability. It may, however, invite other teams to use image fusion and occasionally fiducial markers for the most complex hepatic thermal ablation procedures. Beyond the facilitation of the gesture itself, we were able to note a possible widening of the indications, with virtually no cases remaining ineligible to PTA due to poor tumor visualization. Second, the cohort included a heterogeneous group of tumors (HCC and metastases from different origins), potentially influencing detectability and results. Third, the relatively small sample size introduces the risk of bias. Fourth, the study did not assess oncological outcomes such as local control or survival, as its objective was limited to evaluating the feasibility and accuracy of fiducial marker placement. Finally, in some cases, fiducial placement failed or lesions remained undetectable, underscoring the technical challenges that remain and the need for further prospective studies.

## Conclusion

Marking invisible or poorly conspicuous hepatic lesions with a fiducial marker under US guidance with fusion technology is safe and feasible with good precision. Our work suggests that routine fiducial marker marking of those occult liver lesions may make them more suitable for percutaneous ablation, potentially widening the indication of PTA to classically hard-to-treat lesions.

## Data Availability

The raw data supporting the conclusions of this article will be made available by the authors, without undue reservation.
